# Antipsychotic adherence patterns and health care utilization and costs among patients discharged after a schizophrenia-related hospitalization

**DOI:** 10.1186/1471-244X-13-246

**Published:** 2013-10-05

**Authors:** Michael Markowitz, Sudeep Karve, Jessica Panish, Sean D Candrilli, Larry Alphs

**Affiliations:** 1 Janssen Scientific Affairs, LLC, Titusville, New Jersey; 2 RTI Health Solutions, Research Triangle Park, North Carolina, USA

**Keywords:** Schizophrenia, Medicaid, Hospitalization, Health care utilization, Adherence

## Abstract

**Background:**

This study aimed to assess antipsychotic adherence patterns and all-cause and schizophrenia-related health care utilization and costs sequentially during critical clinical periods (i.e., before and after schizophrenia-related hospitalization) among Medicaid-enrolled patients experiencing a schizophrenia-related hospitalization.

**Methods:**

All patients aged ≥ 18 years with a schizophrenia-related inpatient admission were identified from the MarketScan Medicaid database (2004–2008). Adherence (proportion of days covered [PDC]) to antipsychotics and schizophrenia-related and all-cause health care utilization and costs were assessed during preadmission (182- to 121-day, 120- to 61-day, and 60- to 0-day periods; overall, 6 months) and postdischarge periods (0- to 60-day, 61- to 120-day, 121- to 180-day, 181- to 240-day, 241- to 300-day, and 301- to 365-day periods; overall, 12 months). Health care utilization and costs (2010 US dollars) were compared between each adjacent 60-day follow-up period after discharge using univariate and multivariable regression analyses. No adjustment was made for multiplicity.

**Results:**

Of the 2,541 patients with schizophrenia (mean age: 41.2 years; 57% male; 59% black) who were identified, approximately 89% were “discharged to home self-care.” Compared with the 60- to 0-day period before the index inpatient admission, greater mean adherence as measured by PDC was observed during the 0- to 60-day period immediately following discharge (0.46 vs. 0.78, respectively). The mean PDC during the overall 6-month preadmission period was lower than during the 6-month postdischarge period (0.53 vs. 0.69; *P* < 0.001). Compared with the 0- to 60-day postdischarge period, schizophrenia-related health care costs were significantly lower during the 61- to 120-day postdischarge period (mean: $2,708 vs. $2,102; *P* < 0.001); the primary cost drivers were rehospitalization (mean: $978 vs. $660; *P* < 0.001) and pharmacy (mean: $959 vs. $743; *P* < 0.001). Following the initial 60-day period, both all-cause and schizophrenia-related costs declined and remained stable for the remaining postdischarge periods (days 121–365).

**Conclusions:**

Although long-term (e.g., 365-day) adherence measures are important, estimating adherence over shorter intervals may clarify the course of vulnerability to risk and enable clinicians to better design adherence/risk-related interventions. The greatest risk of rehospitalization and thus greater resource utilization were observed during the initial 60-day postdischarge period. Physicians should consider tailoring management and treatment strategies to help mitigate the economic and humanistic burden for patients with schizophrenia during this period.

## Background

Schizophrenia is a chronic, severe mental illness characterized by psychosis, hallucinations, delusions, and disorganized speech and behavior. In the United States, approximately 2.4 million (1.1%) adults have schizophrenia in any given year [1]. The mortality rate in patients with a schizophrenia diagnosis is 2 to 4 times greater than in the general population [2,3]. Additionally, increased rates of comorbidities, including cardiovascular disorders, diabetes mellitus, hypertension, hyperlipidemia, and other mental illnesses (e.g., anxiety, depression, substance abuse), are often reported among patients with schizophrenia [2,4-6]. These high rates of morbidity and mortality are associated with high treatment management costs among patients with schizophrenia. Estimates suggest that schizophrenia-related direct medical and indirect costs in the United States are approximately $62 billion annually [7].

Antipsychotics are the mainstay in managing acute psychotic exacerbations and as maintenance therapy to prevent relapse among patients with schizophrenia. Recent studies with multi-year follow-up (over 15 years) suggest that a subset of patients with schizophrenia show periods of recovery (defined as “absence of major symptoms and adequate psychosocial functioning throughout the follow-up year”) while not on antipsychotic medications ([8,9]). Some of the characteristics that were observed among patients’ not requiring chronic antipsychotic therapy were less vulnerability to anxiety and psychosis, better neurocognitive skills, and prior experience with periods of recovery [8]. Even though these studies provided encouraging results for a subset of patients, a majority of patients with schizophrenia (over 60%) were still required to be on antipsychotic therapy during follow-up. Thus, a considerable proportion of patients require chronic antipsychotic use, in which adherence to the prescribed antipsychotic regimen is important. Nonadherence to antipsychotic therapy is significantly associated with schizophrenia-related relapse and hospitalization [10-12]. Among patients hospitalized for a schizophrenia relapse, the likelihood of later rehospitalization is high [13-16]. Furthermore, following discharge from the hospital, patients with schizophrenia often face challenges transitioning into the community. This may result in homelessness, social isolation, unemployment, and imprisonment [17-20].

Following a schizophrenia-related hospital discharge, patients with schizophrenia generally are expected to have an increased risk for rehospitalization and are likely to incur greater health care costs as they transition to community care. A prior study [15] suggested that schizophrenia patients without an outpatient visit within 60 days following hospital discharge had a significantly higher risk for rehospitalization within 90 days of discharge. To address this issue, the National Committee for Quality Assurance, which develops the Healthcare Effectiveness Data and Information Set (HEDIS) performance measures for health plans, proposed a measure assessing the rate of outpatient follow-up among hospital-discharged patients with schizophrenia [21]. The objective of this measure is to assess the percentage of individuals enrolled in Medicaid who have at least one follow-up outpatient visit within 7 and 30 days of discharge from a schizophrenia-related hospitalization. It is anticipated that the improved 7- and 30-day follow-up rates expected from this measure should help lower the rate of rehospitalization, improve overall health outcomes, and lower the economic burden on the state Medicaid programs. Thus, adequate and timely medical follow-up after hospital discharge is important in potentially lowering the rate of readmission and the associated economic burden. However, limited data exist that evaluate antipsychotic adherence patterns, heath care utilization, and the associated economic burden followed sequentially across smaller high-risk postdischarge follow-up periods (i.e., 60 days) among schizophrenia patients. The objective of this study was to assess antipsychotic adherence patterns and all-cause and schizophrenia-related health care utilization and costs sequentially during critical high-risk postdischarge periods (i.e., before and after schizophrenia-related hospitalization) for patients experiencing a schizophrenia-related hospitalization. Findings of this analysis may help with efficient resource allocation and in designing interventions specifically to target this high-risk population.

## Methods

### Study design and data source

This was a retrospective, longitudinal cohort analysis of the MarketScan Medicaid Multi-State database for the years 2004 through 2008. For confidentiality purposes, the researchers were not aware of which US 11 states had contributed data to this database. Data included medical (i.e., hospital inpatient, hospital outpatient, emergency department [ED], physician office, long-term care, and ancillary care) and prescription drug claims for Medicaid-enrolled individuals. The medical claims data included information on date of service, physician diagnosis (up to 15 primary and secondary diagnoses), primary and secondary procedures conducted, length of stay (for inpatient, ED, and long-term care settings only), and charges associated with the claim. The pharmacy claims data included National Drug Code, days’ supply, dosage form, therapeutic class of the drug, and charges associated with the claim. In addition to medical and pharmacy claims, the database also contained an eligibility file that included information on patient demographic characteristics (e.g., age, gender, health coverage, and race) and periods of Medicaid enrollment. Individuals in the medical, pharmacy, and eligibility files could be linked and longitudinally followed using a unique encrypted identification number.

The conduct of this study was approved by the Institutional Review Board at RTI International.

### Patient selection

The following inclusion and exclusion criteria were used in the selection of study cohort:

• Inclusion criteria (all of which must have been met)

– Patients with an inpatient admission with primary schizophrenia diagnosis (i.e., International Classification of Diseases, 9th Revision, Clinical Modification [ICD-9-CM] diagnosis code 295.xx, except 295.x7) between July 1, 2004, and December 31, 2007, and associated hospital discharge on or before December 31, 2007.

– Continuous Medicaid enrollment during the 6-month period before the index admission date (i.e., date of the first observed inpatient admission), hereafter referred to as the “preindex period”; during the index inpatient admission; and during the 12-month period after the index discharge date (i.e., date of discharge associated with the index admission), hereafter referred to as the “postindex period.”

– In addition to the index schizophrenia admission, at least one outpatient or physician office visit with a primary schizophrenia diagnosis or two or more prescription claims for first- or second-generation antipsychotic medications during the preindex and postindex periods.

• Exclusion criteria

– Patients with schizophrenia-related inpatient admissions (secondary diagnosis) during the preindex period. Because prior schizophrenia-related inpatient admission has been associated with a high risk of schizophrenia-related rehospitalization, this exclusion criterion ensured that each selected patient had a uniform-length (i.e., 6 months) clean period during which there was no evidence of a schizophrenia-related inpatient admission.

– Patients with at least one primary diagnosis claim for bipolar or schizoaffective disorder or more than two primary diagnoses claims for unipolar disorder during the postindex period. This criterion was primarily considered to lower the likelihood of misclassification of schizophrenia with other mental health conditions.

– Patients aged 17 years or younger at their index admission date and patients aged 65 years or older at their follow-up end date.

– Patients with dual eligibility (i.e., Medicaid and Medicare) were excluded because patients with coverage under both plans would likely have incomplete claims history in the data recorded for each plan, and as such, certain events may not be reflected in their Medicaid claims record.

– Patients without mental health and substance abuse coverage.

**Figure 1:**
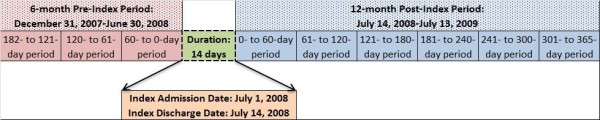
**Schematic representation of study periods**^**a**^**.**

A schematic representation of the study period is included in Figure 1.

### Study measures

#### Antipsychotic adherence patterns

Adherence to antipsychotic medications was evaluated on a shorter-term basis: sequentially over time for 60-day intervals starting 6 months before hospitalization and for up to 12 months after discharge. Adherence also was evaluated on a longer term-basis: comparing the full 6 months before and the full 6 months after index hospitalization discharge. Adherence to antipsychotic treatment was evaluated by the proportion of days covered (PDC) measure [22-24]. The PDC method assesses drug availability for each day of the study period, rather than cumulative exposure as assessed by the commonly used medication possession ratio. Because the study included distinct time intervals of evaluation (e.g., 0- to 60-day and 61- to 120-day periods following the index discharge date), adherence measurement for each day during the study period was considered more appropriate than measuring cumulative adherence. The following formula was used to calculate PDC:

PDC=Totaldaysofdrugavailabilitydays'supplyintheperiodofevaluation÷Numberofdaysintheperiodofevaluation‒Numberofdayshospitalizedduringtheperiodofevaluation.

Medicaid claims data do not include details on medications provided during an inpatient stay. Thus, an assumption was made that patients received a full supply of their antipsychotic medications during a hospital stay and were 100% adherent to their supplied medications during the stay, and days hospitalized were subtracted from the denominator [22,25]. Moreover, because the data did not include details on days’ supply for antipsychotic depot preparations (i.e., risperidone, fluphenazine, and haloperidol) administered in an office setting, the following approved dosage durations were used as a proxy for the days’ supply for these treatments:

• Risperidone long-acting injection: 2 weeks

• Haloperidol long-acting injection: 4 weeks

• Fluphenazine long-acting injection: 4 weeks

Patients with a PDC value of less than 0.8 (i.e., < 80% adherence) were classified as nonadherent, whereas patients with a PDC value of 0.8 or greater were classified as adherent [26]. PDC was assessed for the preindex periods (i.e., 182- to 121-day, 120- to 61-day, and 60- to 0-day periods; and 6 months overall) and postindex periods (i.e., 0- to 60-day, 61- to 120-day, 121- to 180-day, 181- to 240-day, 241- to 300-day, and 301- to 365-day periods; and 12 months overall).

#### Health care utilization and costs

Overall (i.e., all-cause) and schizophrenia-related health care utilization and costs were assessed (both unadjusted and covariate-adjusted analyses) on a shorter-term basis: sequentially over time for 60-day intervals during the 12 months after discharge. Resource use and costs were also evaluated on a longer term-basis: comparing the full 6 months before the index admission and the full 6 months after index hospitalization discharge. Schizophrenia-related utilization was defined as medical claims with a primary diagnosis for schizophrenia (i.e., ICD-9-CM: 295.xx, except 295.7x) or pharmacy claims for first- or second-generation antipsychotics. Both all-cause and schizophrenia-related health care utilization and costs were assessed for various care settings: inpatient, outpatient, physician office, ED, pharmacy, and other ancillary care. Health care utilization was further documented as follows: 1) the percentage of patients with one or more claims for the aforementioned care settings; 2) the number of unique inpatient admissions; 3) the mean number of inpatient days; and 4) other categories of resource use, including the following: number of outpatient, physician office, ED, and other ancillary care visits and pharmacy claims, each assessed separately.

Costs associated with health care utilization were assessed and adjusted to 2010 US dollars, using the medical care component of the Consumer Price Index. In cases where an inpatient stay spanned two or more follow-up time periods, a length of stay (LOS) was assigned to each follow-up period based on the number of days the patient was in the hospital during each period. Similarly, a per-day cost (i.e., total cost for the inpatient episode ÷ LOS) was calculated for each inpatient episode. Costs then were assigned to each follow-up period on the basis of the LOS in that period.

#### Statistical analyses

All statistical analyses were conducted using the SAS (version 9.2) statistical software package (SAS Institute Inc., Cary, North Carolina). Descriptive statistics were generated for all study measures, which included frequency distributions for categorical variables and mean values and standard deviations (SDs) for continuous variables. Paired *t* tests were used to compare univariate (i.e., unadjusted) differences in continuous measures of interest (e.g., the number of inpatient admissions), and McNemar’s test was used for categorical measures of interest (e.g., had an inpatient admission) between each adjacent postindex period. No adjustment was made for multiplicity.

Multivariable regression analyses were conducted to assess differences in all-cause and schizophrenia-related health care utilization and costs between each adjacent postindex period, after adjusting for patient baseline demographic and clinical characteristics. The primary independent variable for this study was an indicator for 60-day follow-up periods. All the study measures were compared between each adjacent 60-day follow-up period, using an indicator for each follow-up period. For example, comparing inpatient costs during the 0- to 60–day period versus 61- to 120–day period, the study period indicator was considered 1 if the study period was 61- to 120-days and 0 if the study period was 0- to 60-days (i.e., reference category).

Other covariates considered for this study include patient characteristics (i.e., age, gender, race, health plan type, basis of Medicaid eligibility, mental health and substance abuse coverage, and index hospitalization discharge status) and the baseline comorbidity burden assessed using the Deyo-adapted Charlson Comorbidity Index score [27]. Finally, prior costs have been shown to predict follow-up heath care utilization and costs [26] and have been previously used as a proxy for disease severity [28,29]. Consequently, the preindex period’s all-cause total health care costs and the sum of preindex period costs were assessed and used as a proxy for disease severity.

The type of regression analysis employed depended on the nature of the outcome that was assessed. For count data (e.g., number of physician office visits), repeated measures Poisson or negative binomial regression models were used. The selection of Poisson or negative binomial regression models was based on the model fit, assessed using a Pearson chi-square test. We estimated incidence rate ratios (IRRs) and corresponding 95% confidence intervals (CIs) for the count data models. IRRs based on these models indicated an increase (IRR > 1) or decrease (IRR < 1) in the rate of utilization of the measure being modeled during a follow-up period, compared with the adjacent follow-up period. Additionally, repeated measures logistic regression models were used for dichotomous measures (e.g., had a physician office visit), where the odds ratio (OR) based on the model indicated the increased (IRR > 1) or decreased (IRR < 1) likelihood of the event during a follow-up period, compared with the adjacent follow-up period.

Finally, for cost outcomes (e.g., all-cause physician office visits costs), repeated measures generalized linear models (GLMs) with a log-link function and gamma distribution were used. In comparison with ordinary linear regression involving log-transformed cost data, GLM methodology offered several advantages, primarily because it estimates covariate-adjusted predicted mean costs on a dollar scale, which then can be compared using the Student *t* test [30,31]. The GLM methodology avoids potential biases resulting from the Duan smearing method for retransforming the predicted coefficient from log-transformed cost data [30,32].

## Results

### Baseline characteristics

Additional file 1 presents the sample attrition chart, and baseline patient characteristics are presented in Table 1. Of the 2,541 patients with schizophrenia who met all study inclusion criteria, 56.7% were males and over 59% were black. The mean (SD) age of the study cohort was 41.17 (12.18) years. A majority (61.3%) of patients were enrolled in a fee-for-service health plan. Over 90% had “blind/disabled individual” as the reason for Medicaid eligibility. Over 88% of the study sample had discharge disposition coded as “discharged to home self-care.”

**Table 1:** Baseline characteristics

**Characteristic**	**All patients**	
Total Sample	2,541	100.00
Gender, n (%)		
Male	1,440	56.67
Female	1,101	43.33
Age at Index Admission Date^a^		
Mean (SD) yrs.	41.17	12.18
Age distribution (years), n (%)		
18-24	315	12.40
25-34	493	19.40
35-44	578	22.75
45-54	763	30.03
55-64	392	15.43
Race/Ethnicity, n (%)		
White	807	31.76
Black	1,509	59.39
Hispanic	16	0.63
Other	209	8.23
Health Coverage, n (%)		
Fee-for-service	1,557	61.28
Capitated	984	38.72
Basis of Medicaid Eligibility, n (%)		
Blind/disabled individual	2,393	94.18
Adult (not based on unemployed status)	69	2.72
Child (not child of unemployed adult, not foster care child)	65	2.56
Other^b^	14	0.55
Hospital Discharge Status, n (%)		
Discharged to home self-care	2,254	88.71
Transfer to skilled nursing facility	47	1.85
Transfer to other facility	122	4.80
Left against medical advice	14	0.55
Other alive status	1	0.04
Not yet discharged/transferred	26	1.02
Missing/unknown	77	3.03
Preindex Period^c^ All-Cause Health Care Costs		
Mean (SD)	$8,162.64	$10,793.97
Preindex Period Depot Antipsychotic Use^d^, n (%)		
Yes	126	4.96
No	2,415	95.04
Preindex Period Charlson Comorbidity Index Score		
Mean (SD)	0.87	1.56

*SD* standard deviation.

^a^The date for the first observed schizophrenia-related inpatient admission defined the index admission date.

^b^Other included foster-care child, aged individual, and eligibility status unknown.

^c^The 6-month period prior to the index admission date defined the preindex period.

^d^Defined as at least 1 claim for depot antipsychotic preparation, including risperidone, fluphenazine, and haloperidol.

### Antipsychotic therapy adherence

The overall 6-month preindex period mean (SD) PDC was lower than the 6-month postindex period mean PDC (0.53 [0.39] vs. 0.69 [0.32]; *P* < 0.001) (Table 2). Similarly, a greater proportion of patients was adherent (PDC ≥ 80%) to antipsychotic therapy during the postindex period than during the preindex period (51.2% vs. 38.6%; *P* < 0.001). Over each adjacent 60-day increment in the 6-month preindex period, a decline in the adherence rate was observed. The mean adherence rate was 63% during the 182- to 121-day period, which declined to 50% during the 121- to 61-day period and to 46% during the 60- to 0-day period (just prior to the index inpatient admission). In contrast to the 60- to 0-day period, the adherence rate considerably improved during the 0- to 60-day period (immediately following index hospital discharge) (46% vs. 78%; *P* < 0.001). However, the adherence rate declined by 15% during the 61- to 120-day postdischarge period, compared with the 0- to 60-day postdischarge period (*P* < 0.001), then remained relatively stable during the remaining follow-up periods (range: 58%-63%).

**Table 2:** **Adherence**^**a **^**to antipsychotic**^**b **^**therapy among medicaid-enrolled schizophrenia patients**

**Adherence**	**Preindex period**							
	**182-121 Days**		**121-61 Days**		**60-0 Days**		**Overall 6 Months**	
PDC^c^								
Mean (SD)	0.63	0.39	0.50	0.44	0.46	0.45	0.53	0.39
PDC Categorical (80% Threshold), n (%)								
Nonadherent total (PDC < 80%)	1,227	48.29	1,483	58.36	1,572	61.87	1,560	61.39
Adherent total (PDC ≥ 80%)	1,314	51.71	1,058	41.64	969	38.13	981	38.61
Total	2,541	100.00	2,541	100.00	2,541	100.00	2,541	100.00
**Adherence**	**Postindex period**							
	**0-60 Days**		**61-120 Days**		**121-180 Days**		**Overall 6 Months**	
PDC^c^								
Mean (SD)	0.78	0.27	0.63	0.40	0.62	0.42	0.69	0.32
PDC Categorical (80% Threshold), n (%)								
Nonadherent total (PDC < 80%)	940	36.99	1,204	47.38	1,183	46.56	1,240	48.80
Adherent total (PDC ≥ 80%)	1,601	63.01	1,337	52.62	1,358	53.44	1,301	51.20
Total	2,541	100.00	2,541	100.00	2,541	100.00	2,541	100.00

*PDC* proportion of days covered, *SD* standard deviation.

^a^Adherence measured during the 6-month period before the index inpatient admission and the 6-month period after the index discharge date.

^b^Antipsychotic therapy included first- and second-generation antipsychotic medications.

^c^PDC = total days of drug availability ÷ (days in study period – days hospitalized).

### Unadjusted health care utilization and costs

Additional files 2 and 3 present details on unadjusted health care utilization and associated costs during the follow-up periods, by care settings. Because health care utilization and costs were considerably higher during the initial 0–60 day period compared with the other 60-day follow-up periods, the following results focus primarily on the 0–60 day versus the 61–120 day period.

### Unadjusted schizophrenia-related utilization and costs

**Figure 2:**
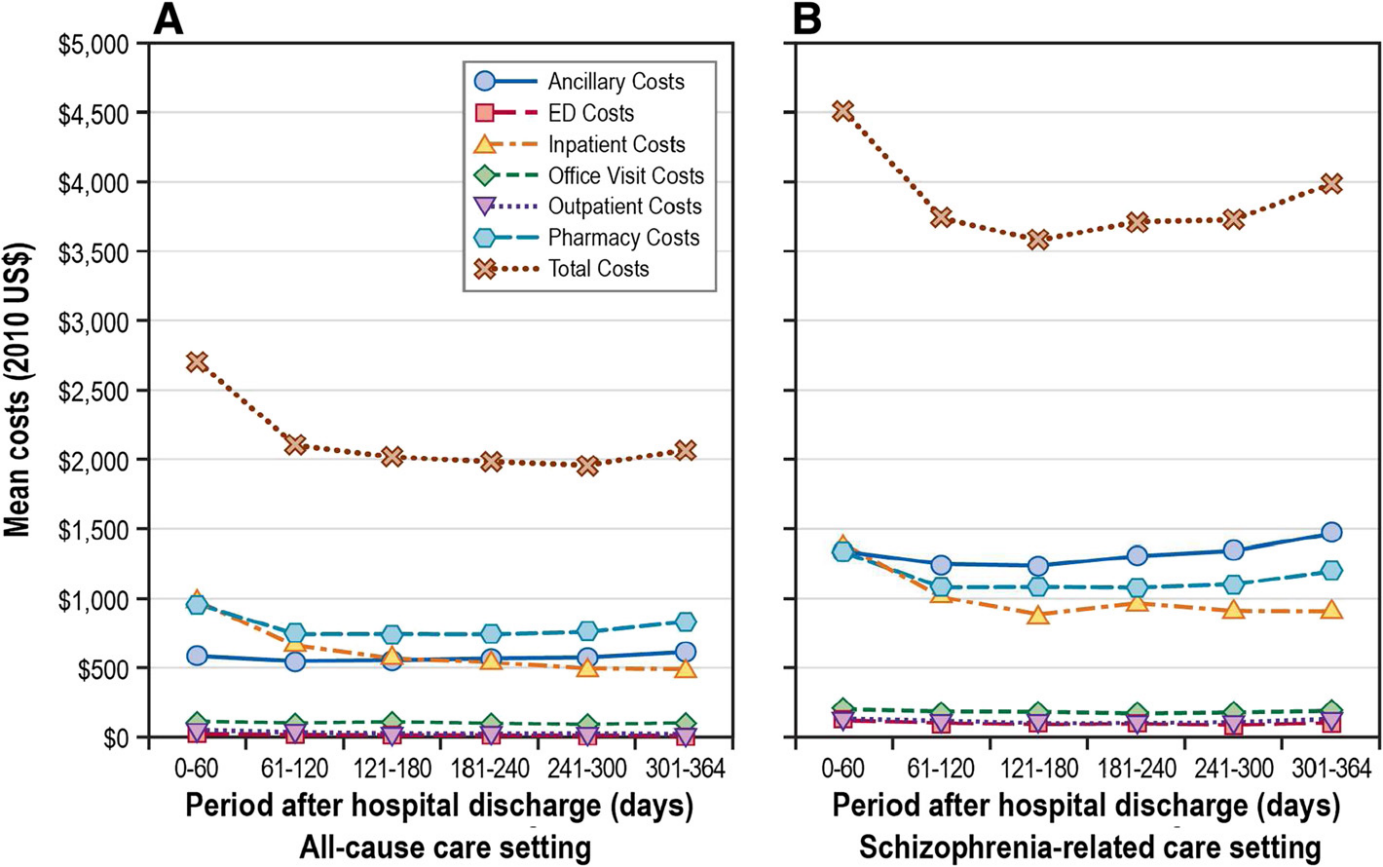
**Follow-up period unadjusted health care costs,**^**a **^**by care setting. A**. Unadjusted all-cause cause health care costs; **B**. Unadjusted Schizophrenia-related health care costs. ED = emergency department. ^a^ The 12-month period following the index discharge date defines the postindex period.

A significantly greater proportion of patients had a schizophrenia-related inpatient admission (13.9% vs. 8.3%; *P* < 0.001) during the 0- to 60-day period than during the 61- to 120-day postdischarge period. The proportion of patients with a schizophrenia-related hospitalization significantly declined during the 61- to 120-day postdischarge period (13.9% vs. 8.3%; *P* < 0.001), compared with the 0- to 60-day postdischarge period; the proportion declined but did not differ statistically during the remaining postdischarge follow-up periods (range: 6.2%-7.9%; *P* > 0.05 for all comparisons) (see Additional file 2). Schizophrenia-related utilization was also significantly greater for other care settings, including hospital outpatient visits (*P* < 0.001), ED visits (*P* = 0.009), and pharmacy claims (*P* < 0.001) during the 0- to 60-day postdischarge period than during the 61- to 120-day postdischarge period. Schizophrenia-related total medical costs were significantly greater during the 0- to 60-day period than during the 61- to 120-day postdischarge period (mean: $2,708 vs. $2,102; *P* < 0.001), with rehospitalization (mean: $978 vs. $660; *P* < 0.001) and pharmacy (mean: $959 vs. $743; *P* < 0.001) costs being the two primary drivers of these increased costs (Figure 2 and Additional file 3). Finally, schizophrenia-related total medical costs (mean: $6,931 vs. $3,442; *P* < 0.001) were significantly greater during the 6-month postdischarge date period compared with the 6-month preindex period.

### All-cause utilization and costs

A significantly greater proportion of patients had an all-cause hospitalization (17.5% vs. 11.3%; *P* < 0.001) during the 0- to 60-day period than during the 61- to 120-day postdischarge period. All-cause hospitalization costs were higher during the initial 0–60 day period (mean: $1,387) compared with the 61–120 day period (mean: $1,008; *P* < 0.001) and remained stable during the remaining follow-up period (range of means: $886-$964; *P* > 0.05 for all comparisons). Similarly, compared with the 0- to 60-day postdischarge period, all-cause costs (mean: $4,514) declined in the 61- to 120-day postdischarge period (mean: $3,738) and remained relatively stable (range of means: $3,577 to $3,984) during the remaining postdischarge periods (see Additional file 3). Finally, all-cause total medical costs (mean: $12,028 vs. $8,163; *P* < 0.001) were significantly greater during the 6-month postdischarge date period compared with the 6-month preindex period, respectively.

### Risk-adjusted health care utilization and costs

**Figure 3:**
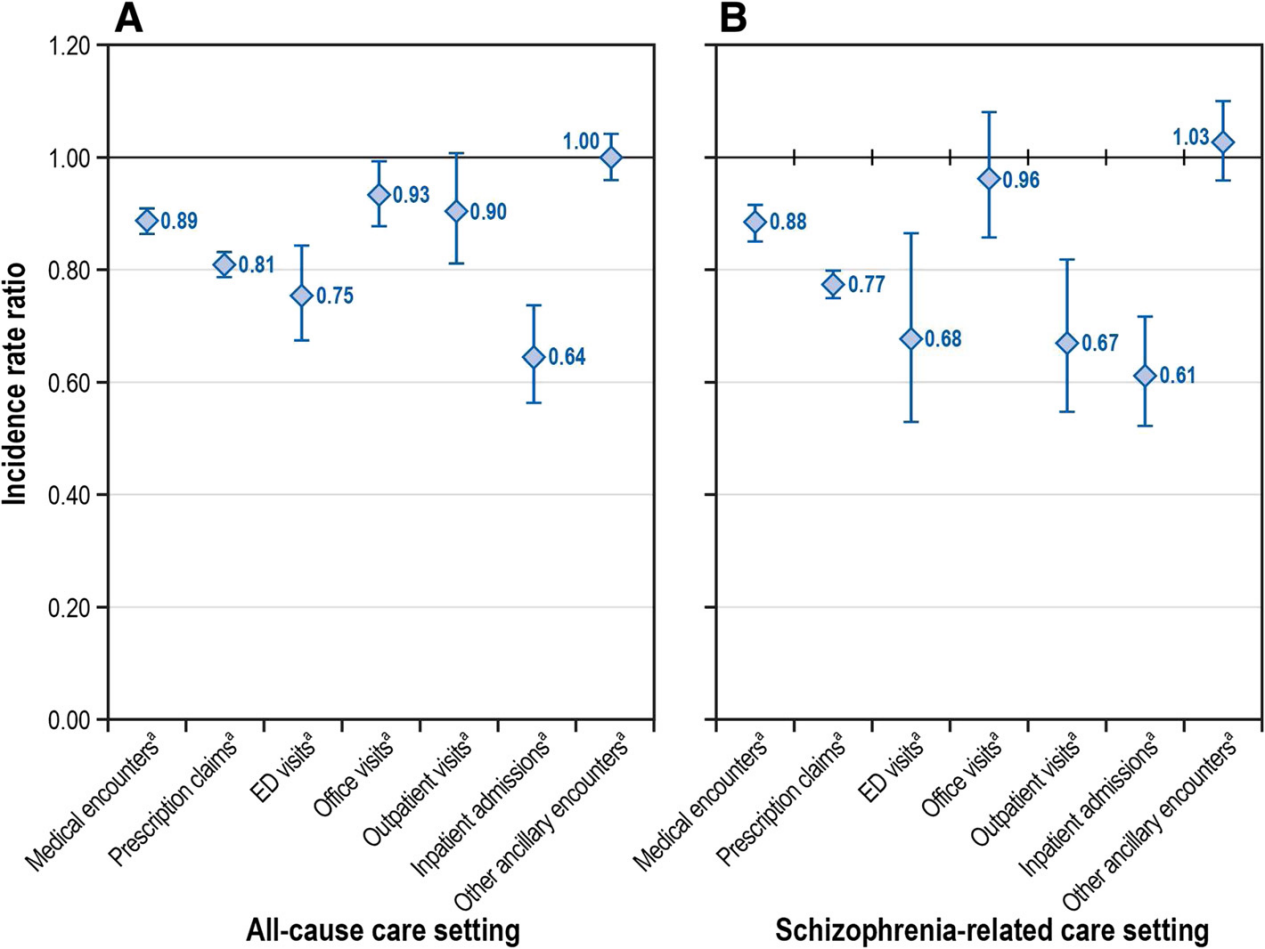
**Risk-adjusted IRRs for 61-120 day period post inpatient discharge, by care setting. A**. IRRs for all-cause cause health care utilization; **B**. IRRs for Schizophrenia-related health care utilization. ED = emergency department; IRR = incident rate ratio. ^a^ IRR based on negative binomial regression model, adjusted for study period (61-120 day period indicator [0-60 day period reference]) and other relevant covariates (i.e., gender, race, age, CCI score, plan type, discharge status, antipsychotic adherence, preindex period health care cost. Graph presents IRR and corresponding 95% confidence intervals).

**Figure 4:**
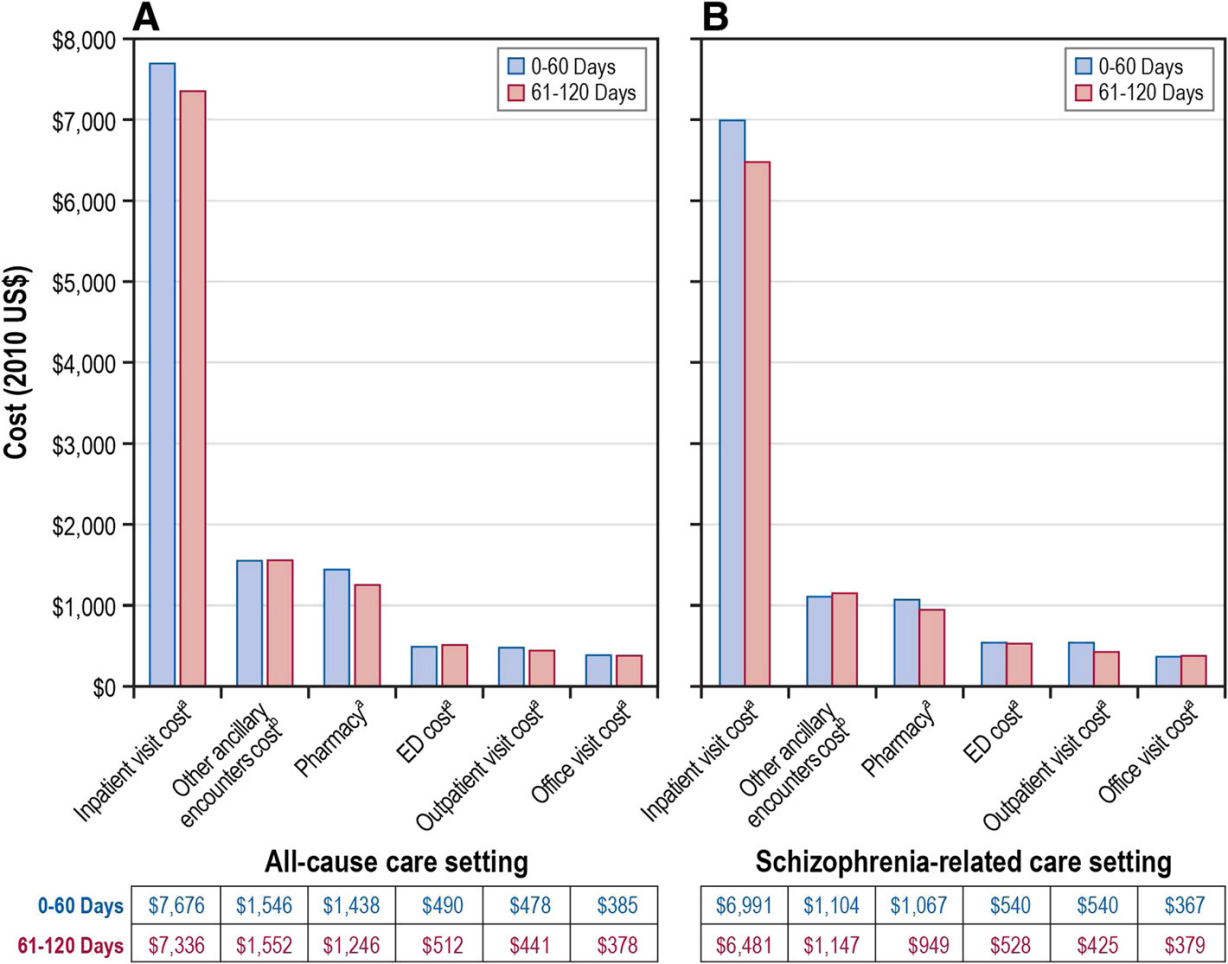
**Adjusted health care costs during 0-60 day and 61-121 day periods post inpatient discharge, by care setting. A**. Adjusted all-cause cause health care costs; **B**. Adjusted Schizophrenia-related health care costs. Predicted costs estimates based on generalized linear models, and corresponding *P* values based on paired *t* test (^a^P < 0.001; ^b^P = 0.039).

Figure 3 presents risk-adjusted IRRs for all-cause and schizophrenia-related health care utilization, by care settings, for the 0- to 60-day and the 61- to 120-day postdischarge periods. Similarly, Figure 4 presents covariate-adjusted predicted all-cause and schizophrenia-related health care costs, by individual care settings, for the 0- to 60-day and the 61- to 120-day postdischarge periods.

### Adjusted schizophrenia-related utilization and costs

The covariate-adjusted rate of schizophrenia-related rehospitalization was significantly lower (IRR: 0.61; 95% CI: 0.52-0.72; *P* < 0.001) during the 61- to 120-day postdischarge period than during the 0- to 60-day postdischarge period (Figure 2). Other covariate-adjusted period comparisons are presented in Additional file 4. The rate of schizophrenia-related rehospitalization did not differ significantly for the remaining postdischarge periods, including 121 to 180 days (IRR: 0.94; *P* = 0.469; compared with 61–120 days), 181 to 240 days (IRR: 0.89; *P* = 0.189; compared with 121–180 days), 241 to 300 days (IRR: 0.93; *P* = 0.420; compared with 181–240 days) and 301 to 364 days (IRR: 0.98; *P* = 0.876; compared with 241–300 days). Schizophrenia-related costs were lower across various care settings, including pharmacy, physician office, hospital outpatient, and inpatient care, during the 61- to 120-day postdischarge period compared with the 0- to 60-day postdischarge period (Figure 4).

### Adjusted all-cause utilization and costs

The all-cause rehospitalization rate was significantly lower (IRR: 0.664; 95% CI: 0.563-0.736; *P* < 0.001) during the 61- to 120-day postdischarge period than during the 0- to 60-day postdischarge period, after adjusting for baseline covariates. After adjusting for covariates, compared with the 0- to 60-day postdischarge period, the predicted all-cause costs were significantly less for various care settings, including pharmacy (mean: $1,438 vs. $1,246; *P* < 0.001), physician office (mean: $385 vs. $378; *P* < 0.001), hospital outpatient (mean: $478 vs. $441; *P* < 0.001), and inpatient (mean: $7,676 vs. $7,336; *P* < 0.001) costs during the 61- to 120-day postdischarge period, respectively.

## Discussion

To the best of our knowledge, this is the first study to assess trends in antipsychotic adherence, health care utilization, and costs sequentially across smaller high-risk postdischarge periods among patients experiencing a schizophrenia-related inpatient admission. A decline in the antipsychotic medication adherence rate from 63% (during the 182- to 121-day preadmission period) to 46% (during the 60- to 0-day preadmission period) prior to the first observed schizophrenia-related hospital admission was observed. The lower antipsychotic adherence of 46% observed during the 60- to 0-days preadmission period prior to the first observed schizophrenia-related hospital admission may have been one of the reasons for the hospital admission. Following hospital discharge, the risk of rehospitalization was significantly greater in the 0- to 60-day postdischarge period than in the 61- to 120-day postdischarge period. However, the risk of rehospitalization did not differ significantly in the remaining postdischarge periods. Similarly, the overall mean schizophrenia-related health care cost for the 12-month postdischarge period was $12,836; over 21% of this amount was incurred in the 0- to 60-day postdischarge period, and approximately 16% was incurred during each remaining 60-day postdischarge period.

Several studies have previously evaluated the association between adherence to antipsychotics and the risk for hospitalization among Medicaid enrollees [33-37]. Findings of these studies suggested that patients nonadherent (< 80%) to antipsychotic therapy have a greater likelihood of hospitalization than adherent patients. In comparison with our study, which assessed adherence at smaller intervals (i.e., 60 days), most of the published studies we found have assessed adherence over a longer follow-up period (e.g., 365 days). Prior study findings suggested that, compared with continuous medication availability, a small gap of 10 days following a missed prescription refill was associated with an increased risk of schizophrenia-related hospitalization (hazard ratio: 1.77; 95% CI: 1.16-2.71) [38]. An earlier study reported that patients with schizophrenia who experienced a therapy gap of 11 to 30 days (OR: 2.8; 95% CI: 1.8-4.6; *P* < 0.001) or 30 days or more (OR: 3.9; 95% CI: 2.5-6.5; *P* < 0.001) had significantly greater risks for hospitalization than did patients without therapy gaps [39]. Similarly, our study, assessing adherence over small intervals, found that adherence to antipsychotics was lower (PDC 46%) during the 60- to 0-day period prior to hospital admission, and this lower adherence may have been one of the factors associated with the hospital admission. The disadvantage of measuring adherence over a long follow-up period is that it masks shorter intervals during which a patient may not be adherent to the prescribed medications and which can result in an inpatient admission. For example, if a nonhospitalized patient refills his or her prescribed medication every 30 days for 10 months (300 days’ drug supply) but then does not refill the medication for the next 2 months, using a 1-year follow-up period in the PDC calculation, the medication adherence rate for this patient would be 82% (= 300 days’ drug supply ÷ 365 days). This patient will be considered adherent for the year, using an 80% threshold, but may be at an increased risk for hospitalization, given the 60-day gap in therapy adherence. With increasing emphasis on identifying high-risk patients for targeted pharmacist interventions (e.g., medication therapy management), measuring medication adherence over smaller intervals may serve as an efficient tool in identifying high-risk patients for these interventions [40].

A second key finding of our study was the identification of a high-risk postdischarge period (i.e., 60 days postdischarge) during which a patient has an increased risk for rehospitalization and incurs greater health care costs. Prior studies have suggested that patients with schizophrenia experiencing hospitalization had a greater likelihood of rehospitalization following hospital discharge, with the first hospitalization event being a significant predictor of the rehospitalization event [13-16]. Consequently, patients experiencing rehospitalization have approximately 5 times greater health care costs (mean: $50,986 vs. $10,352) than patients without a rehospitalization [13]. However, none of these studies have identified the high-risk period(s) during which increased risk of rehospitalization exists. Information about the high-risk period during which the patient has an increased risk for rehospitalization is important from a patient management perspective, primarily because physicians and other health care providers may design treatment strategies for the critical period, which may in turn help reduce the risk of rehospitalization and the associated costs. Our analysis found that the proportion of patients with schizophrenia-related rehospitalization was highest in the 0- to 60-day period (13.9%) following hospital discharge than in any other follow-up 60-day period (range: 6.2%-8.3%). Thus, our findings are an important initial step toward the identification of a high-risk period during which hospital-discharged patients with schizophrenia have a higher risk of rehospitalization and are likely to incur higher health care costs.

Prior studies have defined recovery among hospital-discharged patients with schizophrenia as the absence of positive or negative symptoms and no rehospitalization within the year following hospital discharge [8]. Thus, prevention of rehospitalization evaluated in this study is a critical component of recovery among patients with schizophrenia. Prior research suggests that interventions such as communication between inpatient staff and outpatient physicians’, patients’ initiating outpatient programs prior to discharge, and involvement of patient family during the hospital stay help in the effective transition of patients from inpatient to outpatient care settings and improve clinical outcomes [41,42]. Since hospital-discharged patients with schizophrenia likely differ on various factors such as duration of disease, severity, availability of family and social support, and access to follow-up care, designing a patient-specific discharge and monitoring plan is critical. With our study providing empirical data on the critical period, additional research is required to assess if patient-specific discharge plans and monitoring during this critical period helps reduce the rate of rehospitalization and improve patient recovery.

Furthermore, among patients requiring antipsychotic therapy to control relapses, adherence to the prescribed therapy is critical. This study provides evidence for using short-term adherence as a tool for identifying patients at high risk for an inpatient admission. Interventions such as routine counseling (e.g., pharmacists, nurse, family members) and simplifying dosing regimens (e.g., once-daily, long-acting injectables) may help improve therapy adherence among these high risk patients. For example, a review of interventions aimed to improve adherence to antipsychotics concluded that a combination of educational (e.g., lecture series, drug data sheet upon discharge), behavioral (e.g., weekly group sessions), and affective (e.g., assertive community treatment) interventions helps improve adherence to prescribed therapy [43].

Several limitations must be considered when interpreting these study findings. First, ICD-9-CM diagnosis codes were used in the selection of patients with schizophrenia; coding inaccuracies or misclassification of other mental health conditions (e.g., bipolar disorder) as schizophrenia may lead to misidentification of patients with schizophrenia. However, to reduce the likelihood of misidentification of patients, several measures were undertaken, including 1) the requirement that the patient have at least two medical claims with a diagnosis of schizophrenia or 2) the requirement that the patient have at least one claim with a primary diagnosis of schizophrenia and two prescription claims for antipsychotics, and 3) the exclusion of patients with other similar mental health conditions, including schizoaffective, bipolar, and unipolar disorders. Additionally, prior studies have suggested differences in patient characteristics and outcomes among patients with schizoaffective disorder compared with patients with schizophrenia [44-46]. Thus, separate studies assessing health care utilization and costs during the post-discharge critical period are required in a schizoaffective disorder population rather than considering the common practice of including these patients as a part of schizophrenia population [47,48]. Second, this analysis was limited to Medicaid enrollees, and findings may not be generalizable to individuals enrolled in other federal (e.g., Medicare, Veterans Affairs) or commercial health plans or to individuals without health insurance. Furthermore, several inclusion and exclusion criteria were used to ensure that all the selected patients had adequate and uniform follow-up data, such as requiring patients to have continuous Medicaid enrollment during the entire study period and excluding patients with dual Medicare and Medicaid eligibility and patients without mental health coverage. Because of these criteria, a subset of Medicaid enrollees was used; hence our findings may not be generalizable to all Medicaid enrollees with schizophrenia. Specifically, a majority (> 50%) of patients were lost either because they did not have continuous Medicaid enrollment during the study period or because they had other psychiatric conditions (schizoaffective, bipolar, and unipolar disorders). The continuous enrollment criterion was imposed to ensure that the selected patients had adequate and uniform in length preindex and postindex follow-up data. Similarly, patients with other similar psychiatric conditions were excluded because presence of these conditions would in itself be associated with greater risk of rehospitalization; thus, an attempt was made to limit the study population to a clean schizophrenia patient cohort. Third, a Medicaid payer’s perspective was considered in assessing direct cost estimates; direct medical costs were calculated using the amount paid by Medicaid to service providers for the delivery of services. Thus, the data did not account for patients’ out-of-pocket expenses (e.g., co-insurance, copayment), which would have the effect of underestimating the total direct cost burden for the study cohort. Moreover, indirect costs such as productivity losses and caregiver costs were not assessed; therefore, additional research is required to estimate the indirect cost burden for various clinically relevant postdischarge periods among patients with a schizophrenia-related inpatient admission. Fourth, assessment of adherence to prescribed therapy using an administrative claims database is based on the “days supply” recorded on outpatient pharmacy claims. However, these pharmacy claims only represent prescriptions filled and do not indicate if the medication was consumed. Additionally, these data do not include details on any medication samples received by the patient at a physician office, which may result in underestimation of medication adherence. Finally, the MarketScan Medicaid Multistate database did not include information on certain clinical and demographic factors, such as medication-related adverse events, employment status, education level, income, and receipt of other interventions such as psychoeducation, or psychosocial rehabilitation which may have an effect on access to medical care, adherence to prescribed therapy and resource utilization and costs.

## Conclusions

This study provides insights into two key aspects of patients experiencing schizophrenia-related hospitalization. First, assessing adherence to antipsychotic medications over smaller intervals may serve as a helpful tool in identifying patients at increased risk for hospitalization. Identifying these high-risk patients and designing adherence-related interventions may help reduce the likelihood of hospital admissions and in turn may reduce the associated downstream costs. Second, our study findings suggest that hospital-discharged schizophrenia patients are at an increased risk for rehospitalization during the initial 60-day period as they transition to the community setting, which in turn exerts a greater economic burden on the Medicaid system. Past studies have suggested that restrictions on length of hospital stay as a cost-containment measure increased the likelihood of rehospitalization within 60 days of discharge among mental health patients [49,50]. However, further research is required to better understand additional factors associated with increased health care resource consumption during this initial 60-day period following discharge and may aid physicians in crafting tailored management and treatment strategies to help mitigate the economic and humanistic burden of illness in patients with schizophrenia.

## Abbreviations

CI: Confidence interval; ED: Emergency department; GLM: Generalized linear model; HEDIS: Healthcare Effectiveness Data and Information Set; ICD-9-CM: International Classification of Diseases, 9th Revision, Clinical Modification; IRR: Incidence rate ratio; LOS: Length of stay; OR: Odds ratio; PDC: Proportion of days covered; SD: Standard deviation; US: United States; USD: US dollars.

## Competing interests

Sudeep Karve and Sean Candrilli are employees of RTI Health Solutions, an independent contract research organization that received research funding from Janssen Scientific Affairs, LLC, for this study. Michael Markowitz, Larry Alphs, and Jessica Panish are employees of Janssen Scientific Affairs, LLC.

## Authors’ contributions

MM and LA were involved in the study conceptualization. SK, MM, JP, and SC were the primary developers of the study design. As principal investigator, SK had full access to all the data in the study and takes responsibility for the integrity of the data and the accuracy of the data analysis. SK and SC led all statistical analyses. SK also served as the primary writer in drafting the manuscript text and in interpreting the findings. JP, MM, LA, and SC assisted in interpreting the study findings and drafting the manuscript text; they also served as the primary reviewers of the manuscript. All authors were responsible for approving the manuscript and its contents.

## Authors’ information

Dr. Michael Markowitz was an employee of Janssen Scientific Affairs LLC at the time of conduct of this study.

## Pre-publication history

The pre-publication history for this paper can be accessed here:

http://www.biomedcentral.com/1471-244X/13/246/prepub

## Supplementary Material

Additional file 1 Attrition chart. Click here for file

Additional file 2 **Summary of unadjusted all-cause, and Schizophrenia-related health care utilization during 12-month Postindex period**^**a**^**.** Click here for file

Additional file 3 **Summary of all-cause, and Schizophrenia-related costs during 12-month Postindex period**^**a**^**.** Click here for file

Additional file 4 Rate of events: covariate-adjusted poisson or negative binomial regression results, by study period. Click here for file
