# Supplementary material for: Antipsychotic adherence patterns and health care utilization and costs among patients discharged after a schizophrenia-related hospitalization
Source: BMC Psychiatry. 2013 Oct 5;13:246. doi: 10.1186/1471-244X-13-246 (PMC3853885; doi:10.1186/1471-244X-13-246)
Supplement: Additional file 1 — Attrition chart. [file 1471-244X-13-246-S1.pdf]

Additional File 1. Attrition Chart

Step 1: Patients with schizophrenia-related inpatient admission during July 1, 2004- December 31, 2007 (date of the first observed inpatient admission defines the "index admission date" and the date of discharge associated with the index admission defines the "index discharge date")

N = 13,572

↓

Step 2: Excluding patients with schizophrenia-related inpatient admissions (secondary) during the 6-month period prior to the index admission date

N = 12,188

↓

Step 3: Patients with at least one outpatient or physician office visit or two or more prescription claims for first- or second-generation antipsychotic medications during the 6-month period prior to the index admission date or 12-month period after the index discharge date

N = 11,434

↓

Step 4: Excluding patients with at least 1 primary diagnosis claim (i.e., inpatient, outpatient, physician office, ED, long-term care) for bipolar or schizoaffective disorder, or ≥ 2 primary diagnosis claims for unipolar disorder during 12-month period after the index discharge date

N = 8,636 (after excluding schizoaffective disorder diagnosed patients)

N = 7,840 (after excluding bipolar disorder diagnosed patients)

N = 7,363 (after excluding unipolar disorder diagnosed patients)

↓

Step 5: Patients with continuous Medicaid enrollment (along with drug coverage) during the 6-month period before the index admission date, during the index inpatient admission, and during 12-month period after the index discharge date

N = 3,101

↓

Step 6: Excluding patients aged 18 years or younger at index admission date and patients aged 65 years or older at the follow-up end date (follow-up end date = index discharge date + 364 days)

N = 2,894

↓

Step 7: Excluding patients with dual eligibility (i.e., Medicaid and Medicare)

N = 2,564

↓

Step 8: Excluding patients without mental health coverage

N = 2,541
