# Supplementary material for: Antipsychotic adherence patterns and health care utilization and costs among patients discharged after a schizophrenia-related hospitalization
Source: BMC Psychiatry. 2013 Oct 5;13:246. doi: 10.1186/1471-244X-13-246 (PMC3853885; doi:10.1186/1471-244X-13-246)
Supplement: Additional file 2 — Summary of unadjusted all-cause, and Schizophrenia-related health care utilization during 12-month Postindex perioda. [file 1471-244X-13-246-S2.pdf]

Additional File 2. Summary of Unadjusted All-cause, and Schizophrenia-Related Health Care Utilization During 12-Month Postindex Period[a]

	Study Period											
	12-Month Postindex Period											
	0-60 Days		61-120 Days		121-180 Days		181-240 Days		241-300 Days		301-364 Days	
Overall Health Care Utilization												
Had ≥ 1 encounter, n (%)	2,460	96.81	2,301	90.55	2,296	90.36	2,288	90.04	2,289	90.08	2,314	91.07
Had ≥ 1 schizophrenia-related encounter, n (%)	2,144	84.38	1,869	73.55	1,856	73.04	1,805	71.04	1,799	70.80	1,823	71.74
Number of encounters, mean (SD)	20.12	16.36	17.62	16.37	17.53	16.36	17.63	16.60	17.66	16.63	19.03	17.92
Number of psychiatric-related encounters, mean (SD)	10.16	10.52	8.72	10.97	8.66	10.92	8.60	11.08	8.47	10.91	9.07	11.75
Number of schizophrenia-related encounters, mean (SD)	8.17	9.88	7.15	10.39	7.20	10.40	7.21	10.65	7.06	10.41	7.55	11.25
Pharmacy Utilization												
Had ≥ 1 pharmacy claim, n (%)	2,372	93.35	2,116	83.27	2,069	81.42	2,083	81.98	2,058	80.99	2,098	82.57
Had ≥ 1 schizophrenia-related pharmacy claim, n (%)	2,271	89.37	1,903	74.89	1,869	73.55	1,833	72.14	1,813	71.35	1,846	72.65
Number of pharmacy claims, mean (SD)	9.99	9.15	8.01	8.00	7.94	8.16	7.97	8.13	8.03	8	8.59	8.87
Number of schizophrenia-related pharmacy claims, mean (SD)	3.04	2.74	2.27	2.41	2.22	2.34	2.18	2.34	2.16	2	2.33	2.48

	Study Period											
	12-Month Postindex Period											
	0-60 Days		61-120 Days		121-180 Days		181-240 Days		241-300 Days		301-364 Days	
ED Utilization												
Had ≥ 1 ED visit, n (%)	619	24.36	483	19.01	491	19.32	464	18.26	443.00	17.43	468.00	18.42
Had ≥ 1 schizophrenia-related ED visit, n (%)	152	5.98	111	4.37	107	4.21	96	3.78	81.00	3.19	68.00	2.68
Number of ED visits, mean (SD)	0.41	0.99	0.32	0.85	0.32	0.87	0.30	0.89	0.30	1	0.31	0.93
Number of schizophrenia-related ED visits, mean (SD)	0.07	0.36	0.05	0.27	0.05	0.28	0.05	0.29	0.04	0	0.03	0.24
Physician Office Utilization												
Had ≥ 1 office visit, n (%)	1,341	52.77	1,290	50.77	1,280	50.37	1,295	50.96	1,300.00	51.16	1,358.00	53.44
Had ≥ 1 schizophrenia-related office visit, n (%)	476	18.73	413	16.25	414	16.29	415	16.33	416.00	16.37	402.00	15.82
Number of office visits, mean (SD)	2.01	4.42	1.87	4.22	1.92	4.28	1.89	4.22	1.93	422.00	2.10	4.56
Number of schizophrenia-related office visits, mean (SD)	0.93	3.71	0.88	3.78	0.95	3.84	0.91	3.81	0.91	382.00	0.99	4.24

	Study Period											
	12-Month Postindex Period											
	**0-60 Days**		**61-120 Days**		**121-180 Days**		**181-240 Days**		**241-300 Days**		**301-364 Days**	
Hospital Outpatient Utilization												
Had ≥ 1 outpatient visit, n (%)	702	27.63	645	25.38	623	24.52	597	23.49	585.00	23.02	584.00	22.98
Had ≥ 1 schizophrenia-related outpatient visit, n (%)	251	9.88	189	7.44	164	6.45	145	5.71	132.00	5.19	130.00	5.12
Number of outpatient visits, mean (SD)	0.59	1.59	0.54	1.49	0.50	1.45	0.48	1.37	0.47	135.00	0.52	1.58
Number of schizophrenia-related outpatient visits, mean (SD)	0.20	1.02	0.14	0.80	0.13	0.74	0.11	0.68	0.10	58.00	0.10	0.64
Inpatient Utilization												
Had ≥ 1 inpatient stay, n (%)	444	17.47	288	11.33	269	10.59	245.00	9.64	243.00	9.56	242.00	9.52
Had ≥ 1 schizophrenia-related inpatient stay, n (%)	352	13.85	211	8.30%	201	7.91	171.00	6.73%	160.00	6.30%	158.00	6.22
Number of unique inpatient admissions, mean (SD)	0.21	0.49	0.13	0.39	0.13	0.39	0.12	0.42	0.12	0.39	0.12	0.40
Number of inpatient days, mean (SD)	1.85	5.66	1.40	5.37	1.38	5.72	1.27	5.52	1.24	5.52	1.25	5.39
Number of unique schizophrenia-related inpatient admissions, mean (SD)	0.16	0.42	0.09	0.33	0.09	0.32	0.08	0.32	0.07	0.30	0.07	0.30
Schizophrenia-related number of inpatient days, mean (SD)	1.48	5.15	1.07	4.60	1.11	5.33	1.00	5.02	0.90	4.93	0.91	4.84

	Study Period											
	12-Month Postindex Period											
	**0-60 Days**		**61-120 Days**		**121-180 Days**		**181-240 Days**		**241-300 Days**		**301-364 Days**	
Other Ancillary Care Utilization												
Had ≥ 1 other ancillary care encounter, n (%)	2,016	79.34	1,895	74.58	1,897	74.66	1,889	74.34	1,878	73.91	1,907	75.05
Had ≥ 1 schizophrenia-related other ancillary care encounter visit, n (%)	1,209	47.58	1,098	43.21	1,122	44.16	1,104	43.45	1,099	43.25	1,102	43.37
Number of other ancillary care encounters, mean (SD)	6.92	9.79	6.75	10.46	6.73	10.29	6.87	10.63	6.82	11	7.39	11.24
Number of schizophrenia-related other ancillary care encounters, mean (SD)	3.76	7.99	3.71	8.46	3.76	8.54	3.88	8.85	3.77	9	4.03	9.18

ED = emergency department; SD = standard deviation.

[a] The 12-month period following the index discharge date defines the postindex period.
