# Supplementary material for: Antipsychotic adherence patterns and health care utilization and costs among patients discharged after a schizophrenia-related hospitalization
Source: BMC Psychiatry. 2013 Oct 5;13:246. doi: 10.1186/1471-244X-13-246 (PMC3853885; doi:10.1186/1471-244X-13-246)
Supplement: Additional file 3 — Summary of all-cause, and Schizophrenia-related costs during 12-month Postindex perioda. [file 1471-244X-13-246-S3.pdf]

Additional File 3. Summary of All-cause, and Schizophrenia-Related Costs During 12-Month Postindex Period[a]

	Study Period											
	12-Month Postindex Period											
	0-60 Days		61-120 Days		121-180 Days		181-240 Days		241-300 Days		301-364 Days	
Overall Health Care Costs												
Total all-cause health care costs (US $), mean (SD)	$4,514	$5,447	$3,738	$5,232	$3,577	$4,663	$3,713	$5,649	$3,726	$5,173	$3,984	$5,468
Total schizophrenia-related health care costs (US $), mean (SD)	$2,708	$3,955	$2,102	$3,351	$2,018	$2,998	$1,983	$3,120	$1,957	$3,128	$2,068	$3,073
Pharmacy Costs												
Total pharmacy costs (US $), mean (SD)	$1,332	$1,222	$1,079	$1,125	$1,083	$1,213	$1,078	$1,190	$1,102	$1,266	$1,194	$1,355
Total schizophrenia-related pharmacy costs (US $), mean (SD)	$959	$971	$743	$865	$744	$907	$742	$902	$759	$921	$832	$1,007
ED Costs												
Total all-cause ED costs (US $), mean (SD)	$122	$418	$100	$355	$91	$309	$95	$371	$87	$337	$101	$397
Total schizophrenia-related ED costs (US $), mean (SD)	$22	$137	$16	$106	$13	$91	$12	$83	$12	$107	$9	$86
Physician Office Costs												
Total all-cause office visit costs (US $), mean (SD)	$200	$612	$184	$622	$182	$610	$170	$532	$177	$659	$188	$599
Total schizophrenia-related office visit costs (US $), mean (SD)	$114	$536	$101	$522	$110	$541	$99	$472	$92	$449	$102	$495

	Study Period											
	12-Month Postindex Period											
	0-60 Days		61-120 Days		121-180 Days		181-240 Days		241-300 Days		301-364 Days	
Hospital Outpatient Costs												
Total all-cause outpatient visit costs (US $), mean (SD)	$133	$597	$118	$549	$100	$506	$101	$516	$107	$580	$131	$634
Total schizophrenia-related outpatient visit costs (US $), mean (SD)	$50	$400	$33	$308	$25	$272	$23	$273	$25	$294	$21	$262
Inpatient Costs												
Total all-cause inpatient costs (US $), mean (SD)	$1,387	$4,147	$1,008	$3,998	$886	$3,206	$964	$4,328	$910	$3,509	$907	$3,530
Total schizophrenia-related inpatient costs (US $), mean (SD)	$978	$3,336	$660	$2,674	$569	$2,197	$538	$2,326	$494	$2,249	$489	$2,096
Other Ancillary Care Costs												
Total all-cause other ancillary care encounter costs (US $), mean (SD)	$1,338	$2,593	$1,248	$2,521	$1,236	$2,491	$1,305	$2,756	$1,343	$2,892	$1,464	$3,095
Total schizophrenia-related other ancillary care encounter costs (US $), mean (SD)	$586	$1,446	$549	$1,403	$556	$1,439	$569	$1,444	$576	$1,527	$614	$1,622

ED = emergency department; SD = standard deviation; US = United States.

[a] The 12-month period following the index discharge date defines the postindex period.
