# Supplementary material for: Antipsychotic adherence patterns and health care utilization and costs among patients discharged after a schizophrenia-related hospitalization
Source: BMC Psychiatry. 2013 Oct 5;13:246. doi: 10.1186/1471-244X-13-246 (PMC3853885; doi:10.1186/1471-244X-13-246)
Supplement: Additional file 4 — Rate of events: covariate-adjusted poisson or negative binomial regression results, by study period. [file 1471-244X-13-246-S4.pdf]

Additional File 4. Rate of Events: Covaraite-Adjusted Poisson or Negative Binomial Regression Results, by Study Period

Event Setting	Study Period[a]											
	121-180 Days (vs. 61-120 Days)			181-240 Days (vs. 121-180 Days)			241-300 Days (vs. 181-240 Days)			301-364 Days (vs. 241-300 Days)		
	IRR[b]	95% CI		IRR[b]	95% CI		IRR[b]	95% CI		IRR[b]	95% CI	
Number of total medical encounters	0.982	0.959	1.006	1.002	0.978	1.026	1.005	0.980	1.030	1.085	1.059	1.112[c]
Number of schizophrenia-related total medical encounters	1.006	0.973	1.040	0.986	0.951	1.022	0.988	0.949	1.028	1.071	1.029	1.114[c]
Number of prescription claims	0.968	0.941	0.995[c]	0.996	0.968	1.025	1.008	0.979	1.037	1.074	1.044	1.105[c]
Number of schizophrenia-related prescription claims	0.958	0.929	0.988[c]	0.974	0.945	1.005	0.995	0.966	1.026	1.077	1.042	1.112
Number of ED visits	0.998	0.888	1.122	0.940	0.836	1.056	1.001	0.889	1.128	1.024	0.908	1.155
Number of schizophrenia-related ED visits	1.009	0.790	1.288	0.890	0.670	1.184	0.877	0.642	1.197	0.785	0.556	1.110
Number of office visits	1.024	0.974	1.077	0.992	0.945	1.042	1.027	0.974	1.082	1.089	1.035	1.146[c]
Number of schizophrenia-related office visits	1.112	1.007	1.228[c]	0.956	0.874	1.045	1.061	0.962	1.171	1.034	0.930	1.151
Number of outpatient visits	0.913	0.837	0.996[c]	0.971	0.884	1.067	0.950	0.857	1.054	1.106	1.002	1.221[c]
Number of schizophrenia-related outpatient visits	0.911	0.774	1.072	0.908	0.739	1.117	0.853	0.653	1.112	0.982	0.796	1.211
Number of inpatient admissions	0.961	0.826	1.117	0.955	0.817	1.116	0.958	0.822	1.115	1.020	0.869	1.196

Event Setting	Study Period[a]											
	121-180 Days (vs. 61-120 Days)			181-240 Days (vs. 121-180 Days)			241-300 Days (vs. 181-240 Days)			301-364 Days (vs. 241-300 Days)		
	IRR[b]	95% CI		IRR[b]	95% CI		IRR[b]	95% CI		IRR[b]	95% CI	
Number of schizophrenia-related inpatient admissions	0.937	0.785	1.118	0.885	0.738	1.062	0.926	0.769	1.116	0.984	0.802	1.207
Number of ancillary care encounters	0.996	0.960	1.033	1.014	0.977	1.052	1.006	0.967	1.046	1.095	1.057	1.135[c]
Number of schizophrenia-related ancillary care encounters	1.031	0.975	1.089	1.011	0.952	1.073	0.993	0.930	1.060	1.076	1.016	1.140[c]

CI = confidence interval; IRR = incidence rate ratio; ED = emergency department.

[a] 12-month period following the index discharge date defines the postindex period.

[b] Incidence rate ratio based on negative binomial or Poisson regression model, adjusted for study period and other covariates (i.e., gender, race, age, Charlson Comorbidity Index score, plan type, discharge status, antipsychotic adherence, preindex period health care cost).

[c] Significant at level 0.05.
